# RETRACTION: Comprehensive Analysis of Risk Factors for Postoperative Wound Infection Following Radical Mastectomy in Breast Cancer Patients

**DOI:** 10.1111/iwj.70497

**Published:** 2025-04-02

## Abstract

**RETRACTION**: HuY. , MaoZ. , and XuY. , “Comprehensive Analysis of Risk Factors for Postoperative Wound Infection Following Radical Mastectomy in Breast Cancer Patients,” International Wound Journal 21, no. 4 (2024): e14848, 10.1111/iwj.14848.38578050 PMC10996372

The above article, published online on 05 April 2024, in Wiley Online Library (http://onlinelibrary.wiley.com/), has been retracted by agreement between the journal Editor in Chief, Professor Keith Harding; and John Wiley & Sons Ltd. Following an investigation by the publisher, all parties have concluded that this article was accepted solely on the basis of a compromised peer review process. The editors have therefore decided to retract the article. The authors did not respond to our notice regarding the retraction.

**RETRACTION**: Y. Hu , Z. Mao , and Y. Xu , “Comprehensive Analysis of Risk Factors for Postoperative Wound Infection Following Radical Mastectomy in Breast Cancer Patients,” International Wound Journal 21, no. 4 (2024): e14848, 10.1111/iwj.14848.38578050 PMC10996372

The above article, published online on 05 April 2024, in Wiley Online Library (http://onlinelibrary.wiley.com/), has been retracted by agreement between the journal Editor in Chief, Professor Keith Harding; and John Wiley & Sons Ltd. Following an investigation by the publisher, all parties have concluded that this article was accepted solely on the basis of a compromised peer review process. The editors have therefore decided to retract the article. The authors did not respond to our notice regarding the retraction.

